# Advances in Tissue Engineering of the Temporomandibular Joint Disc: An Overview of Current Status and Future Directions

**DOI:** 10.1155/2022/9696378

**Published:** 2022-07-22

**Authors:** Ashutosh Kumar Singh, Nikita Khanal, Rajib Chaulagain

**Affiliations:** ^1^Department of Oral and Maxillofacial Surgery, Tribhuvan University Teaching Hospital, Maharajgunj Medical Campus, Institute of Medicine, Kathmandu, Nepal; ^2^Ek Ek Paila Foundation, Kathmandu, Nepal; ^3^Department of Oral Biology, Chitwan Medical College, Bharatpur, Nepal

## Abstract

Advances in tissue engineering have progressed to potentially offer a solution to temporomandibular joint disc (TMJ) disorders not amenable to conservative therapies. Conclusive treatment options for patients with end-stage disc disorders requires discectomy and reconstruction of the articular disc with various materials. Tissue engineering TMJ disc is a promising alternative to the limited and sometimes inadequate clinical options in the management of such disorders. However, tissue engineering is far from completion for the TMJ disc regeneration. This review briefly discusses the properties of native disc, the mechanism by which TMJ disorders manifest, and how a tissue engineered disc could assuage the problems inherent in the management of such disorders. Furthermore, the review addresses and provides updates to relevant themes of tissue engineering in regards to the TMJ disc, namely, the scaffolds, cells and biomarkers, hurdles in tissue engineering of the disc, and its application in translation to the clinical practice and future directions.

## 1. Introduction

The temporomandibular joint (TMJ) is a synovial joint between the mandibular condyle and the glenoid fossa of the cranium. The temporomandibular disc is a fibrocartilaginous tissue with a biconcave shape that allows it to fit the bony anatomy and divide the joint into a superior and inferior compartment [1]. It is viscoelastic because of its composition of water (73%) and ground substance (glycosaminoglycans) in a thick collagen matrix, which allows it to expel and imbibe fluid during compression and relaxation, thus allowing smooth loading and unloading of the joint [2]. At the same time, it allows frictionless motion and maintains joint stability and congruity during motion. Morphologically, it has three distinct zones, where the anterior and posterior bands are thicker (4 mm) compared to the thinnest intermediate zone (1 mm) [2].

In a normally functioning TMJ, the continuous wear and tear of joint surfaces and the disc are repaired by the reparative cells present in the synovial fluids and the joint surfaces, but this intricate and delicate balance is oftentimes disrupted by unusual loading due to micro or microtrauma to the joint, the result of which is displacement, elongation, and fragmentation of the disc which bears the brunt of initial forces before it gives way and the joint surfaces are exposed to trauma [3]. Symptoms of disc disorders like clicking, pain on chewing, and difficulty opening the mouth are very common in adults around the world, and most of these are managed by noninvasive techniques like physiotherapy, diet restrictions, pharmacotherapeutic agents, splints, topical agents, lasers, and lavage [4–6]. The prevalence of TMDs has been reported to be anywhere between 6 and 93%, a majority of which is asymptomatic clicking [7–9]. The severe symptoms of trismus along with pain during mouth opening and chewing is reported by around 3–7% of patients, which typically occurs after the disc is perforated, and there are osteoarthritic changes in the condylar head of the mandible. These severe symptoms not amenable to conservative procedures require surgical interventions, the endpoint of which is discectomy and reconstruction with one of available alloplastic or autogenous grafts [10]. Alloplasts have fallen out of favor because they fragment under load and incite foreign body reactions, so the current favorites are the local pedicled temporalis myofascial flap (TMF) [11, 12], the pedicled buccal fat pad flap (PBFP) [13], and free dermis fat graft (DFG) [14–16]. These grafts all have some lingering complications associated with them; TMF is thin, friable, and may fragment or fibrose, thus contributing to trismus and pain on function, PBFP and DFG gradually lose the transplanted fat volume, and long-term results are not available [10, 17]. Moreover, these grafts necessitate second-site surgery which may result in some donor site morbidity and complications.

Tissue engineering (TE) in its essence tries to solve the inherent problems associated with autogenous grafts, namely, donor site morbidity, the inability of autografts to be a replica of the desired tissue anatomically, morphologically and histologically, and lack of mechanical properties required to rehabilitate the organ to its prediseased functional state [18, 19]. In this brief overview, we will present the latest advances in tissue engineering, a TMJ disc implant, limitations that have hindered its development and current technologies that may assuage the current limitations. We will also discuss results from contemporary preclinical studies reporting real-world outcomes from which clinical implications, applicability in humans, and future trends of its application can be derived.

## 2. Materials and Methods

### 2.1. Search Strategy

We searched PubMed and Google Scholar with the following search algorithm: “tissue engineering” AND “temporomandibular disc. Only recent articles published after 2010 were considered to present recent advances in the field. This is a scoping review to update the scientific advancement on this topic; hence, protocol registration was not performed.

### 2.2. Results

Multiple reviews on the topic were analyzed, each reporting on different themes concerning TMJ disc tissue engineering. Recent in vitro and preclinical animal studies were analyzed which presented outcome data of in vivo TMJ disc implants generated with various materials as scaffolds, cell sources, and signaling biomarkers. These findings are presented according to these themes: scaffolds, cell sources and biomarkers, hurdles in tissue engineering of TMJ disc, and future directions.

## 3. Results

### 3.1. Scaffolds

Scaffolds are natural, synthetic, or combination structures that act as a template and carrier of cells and biosignals for the neoformation of a tissue [20, 21]. An ideal scaffold material is yet to be determined for TMJ disc TE, as various conventional scaffold materials were investigated in in vitro studies and were compromised by their limitations [22].

The first used natural scaffolds were collagen fibrous mesh which showed promising results with spontaneous extracellular matrix (ECM) and cartilage formation but was limited by foreign body reaction around the implant. Fibrin, agar, and alginate gels are used as hydrogel which exhibits excellent water retention capacity and encloses seeded cells uniformly, but lack the mechanical strength, shrink in volume, and rapidly degrade; thus, they are disqualified as an ideal scaffold material for TMJ disc TE [23–25]. Autogenous ECM-based bioscaffolds from other organs like the urinary bladder mucosa (UBM) have shown satisfactory collagen and ECM deposition and adequate function in a 6-month follow-up study; however, it lacks the mechanical properties of the native disc, and a major limitation is a difficulty in obtaining an appropriate pore size which is essential for diffusion-based differentiation of seeded cells [26].

Synthetic materials provide freedom and flexibility, as they can be modified in most parameters, processing capacity, mechanical stability, biocompatibility, biodegradability, pore size, and geometry [27, 28]. An ideal scaffold could be fine-tuned based on available biomechanical studies of the human TMJ disc based on its three-dimensional geometry, histological variations, and mechanical properties. Yet an ideal synthetic scaffold eludes for TMJ TE, not only in part of the limitations inherent in the scaffold materials themselves but also because of the complex and unique composition and biomechanics of the TMJ disc compared to other joints like the knee [29, 30]. PGA (polyglycolic acid) scaffolds can support the growth and differentiation of human umbilical cord cells. However, PGA resorbs rapidly and is mechanically weak for adequate strength. PLLA (poly-L-lactic acid) has a slower degradation rate, and studies showed that seeding with TGF-*β*1 results in improved mechanical properties and higher collagen and ECM deposition compared with the PGA control [27]. PLGA (poly-L-lactic-co-glycolic acid) is versatile enough for the modulation of mechanical properties; nonetheless, a study failed to show good interaction with native TMJ disc collagen [31].

A way forward to compensate for the limitations of individual scaffolds is to fabricate composite scaffolds, utilizing multiple materials, each with different desired properties, to end up with an ideal material close to the native disc [32]. A composite scaffold of natural polymers, chitosan, and alginate cross-linked with calcium chloride (CaCl_2_) allowed cell adhesion and upregulation of fibrocartilage formation. Additionally, the composite scaffolds showed similar storage modulus and elastic response comparable to the native TMJ disc.

The combination of synthetic polymers, PLGA, and polycaprolactone (PCL) seeded with recombinant human bone morphogenetic protein 2 (rhBMP-2), connective tissue growth factor, and TGF-*β*3 can be 3D printed mimicking spatially distributed collagen of TMJ disc. Histologically accurate tissue similar to native TMJ disc with a full recovery of the perforated disc was observed in vivo. A study used PCL, photopolymerized hydrogel polyethylene glycol diacrylate (PEGDA), and a combination of the two materials. The combination material showed the best results, as the slow degradation of PCL was congruent with the slow growth rate of the TMJ disc. Moreover, PCL with its relative rigidity provided adequate mechanical properties which could be advantageous during surgery, whereas PEGDA hydrogel promoted cellular adhesion and lubrication. Thus, a reduction of joint friction and the distribution of the functional load allowed by PEGDA and scaffold geometry and mechanical properties of PC demonstrated that the combination of materials could lead to a desired progress in the TMJ disc TE.

3D printing of scaffolds can achieve high precision and accuracy, with the ability to fabricate complex geometric shapes with close to native spatial cell distribution and mimic the native ECM [19, 33]. A layered fabrication process allows cells and growth factors to be included, which offers better control of desired tissue architecture. Tarafder et al. reported spatiotemporal bioprinting systems with PLGA microspheres and growth factors, seeding mesenchymal stem cells (MSCs) onto the printed scaffold with geometry and contour to a native TMJ disc [34].

In a study using nanoassembly and nanocoating technologies, titanium dioxide surface modification was implemented with TMJ disc cells which showed a proportional increment in cell proliferation and extracellular matrix (ECM) deposition with increasing thickness of nanofilms [35] layered nanoassembly with single or composite scaffold materials such as polycaprolactone (PCL) or polylactic acid (PLA) may enhance the results of the TE disc.

To bypass the complications of scaffold-based TE, the scaffold-free self-assembling process has been reported to generate a functional disc resembling the native disc which is mechanically robust [36]. However, the scaffold-free processes lack the flexibility of scaffold-based approaches like scaffold functionalization with biomolecules. Nevertheless, exogenous stimulation has shown promise with increased mechanical properties without biomolecular signaling [37]. Self-assembled scaffold-free disc implants have approached native values in mechanical properties due to the synergism of biochemical and exogenous mechanical stimuli [38, 39].

In a randomized controlled preclinical trial on interpositional TMJ discs in the black merino sheep model (TEMPOJIMS) [40], the authors compared three 3D tailored TMJ disc implants: polyglycerol sebacate (PGS) scaffold reinforced with electrospun polycaprolactone (PCL) fibers on the outer surface (PGS + PCL), PCL and polyethylene glycol diacrylate (PEGDA) (PCL + PEGDA), and PCL only. None of the implants could regenerate a new autologous disc; however, PGS + PCL was safe and was not observable in multiorgan analysis, demonstrated rapid resorption in 6 months, and prevented further condyle degenerative changes in the sheep TMJ. The PCL + PEGDA and PCL implants showed detrimental changes when compared to the discopexy controls [41]. The results reimpose that we should rather not interpose any material in the joint than to use an unsafe and unreliable material.

### 3.2. Cell Sources and Growth Factors

The success of TMJ-related tissue engineering is dependent upon selection of cell source that are utilized for seeding the scaffolds. The primary cell source in TMJ tissue engineering include autologous TMJ disc cells, articular chondrocytes, costal chondrocytes, and allogenic cells. The native TMJ disc cells have been of great interest of many researchers; however, the challenges associated with healthy tissue such as donor site morbidity and degenerative changes have also been reported [42]. Due to these reasons, other alternatives to TMJ disc cells were explored. In this instance, the use of cocultures of articular chondrocytes and meniscus cells when treated with two exogenous stimuli resulted into formation of collagen fibrils similar native tissue [43]. Further to address the issue of cell scarcity and donor site morbidity, costal chondrocytes were also utilized [44, 45]. To minimize the issue of donor site morbidity, many other options were explored such as use of nonautologous cells. However, even with the use of nonautologous cells, the problem of immunogenicity was still debatable. A ray of hope was then observed with the use of stem cells [46].

Stem cells (mesenchymal and embryonal stem cells) have been successfully used in TMJ tissue engineering. The mesenchymal stem cells (MSCs) have self-renewal property and have capacity for differentiation into various cell lineages. The MSCs have been explored to a great extent for the treatment of medical problems due to their potential role in tissue repair and reduction of inflammation [47]. They have been obtained from many sources for TMJ articular disc tissue engineering including adipose tissue, bone marrow, synovial fluid, muscle, dermis, blood, and dental pulp. Among these, the MSCs derived from TMJ were promising in TMJ tissue engineering as they were associated in repair and regeneration [46].

Notably, MSCs derived from synovial fluid and synovium have stemness trilineage differentiation, self-renewal capacity, and immunosuppressive properties [48]. Both synovial membrane and synovial fluid derived mesenchymal cells isolated from TMJs when induced to proliferate and differentiate in vitro, the cells displayed fibroblast like, spindle-shaped morphology [48]. In addition, these cells differentiated into other cell lineages such as osteogenic, chondrogenic, adipogenic, and neurogenic lineages [49–51]. The MSCs also release growth factors, cytokine, exosomes, and extracellular vesicles. These are called as “secretome” and are said to fluctuate with physiological and pathological conditions. They are said to exert the paracrine effect and also play an important role in transfer of protein, lipid, and genetic material to the recipient cells [52].

The use of type I collagen matrix isolated from rabbit tendon favored the regeneration of TMJ articular disc in rabbit [53]. In another study to observe the changes in the extracellular matrix in partial discectomized rabbit, the reconstituted collagen template favored regeneration of articular disc suggesting the role of type I and type II collagen in regeneration [54]. A unique property of cartilage is that it has limited capacity to repair and small injuries can eventually lead to progressive damage to TMJ. Hence, new approaches, such as use of biological signals, to regenerate and repair the injured cartilage have gained special interest. In case of TMJ tissue engineering, the potential role of biological signals is to activate signaling pathways that initiate the extracellular protein production. The prominent growth factors that regulate growth and function of introduced cells and host cells are fibroblast growth factor 2 (FGF-2), transforming growth factor *β*1 (TGF-*β*1), and insulin like growth factor (IGF) [55].

It has been well documented that laser treatment modulates cellular properties. Photobiomodulation have been used to decrease inflammation and promote wound healing. Photobiomodulation with low-level laser therapy has shown to reduce pain sensation in rats [56]. Studies using photobiomodulation and adult stem cells have shown differentiation of stem cells. Adipose-derived stem cells are component of mesenchymal cell lineage and have qualities to restore and renew tissues [57]. Using photobiomodulation, the differentiation of adipose-derived stem cells into fibroblastic and chondrogenic phenotype was shown suggesting a treatment option for degenerative joint disorders patients [58].

In addition, various growth factors such as bone morphogenetic protein 2 (BMP-2), connective tissue growth factor (CTGF), and platelet-rich plasma (PRP), epidermal growth factor, interleukin 1, and tumor necrosis factor alpha used alone or in combination with cells and/or scaffolds have a role in proliferation and production of collagen and glycosaminoglycans (GAG), and hence, they have been used to regenerate TMJ discal tissues too [31]. Among these growth factors, PRP has been widely used due to the presence of various growth factors in it [59]. In a study to explore the role of PRP, the regeneration of hyaline and fibrocartilage was higher in surgical defects treated with PRP than in the control group [60].

**Figure 1:**
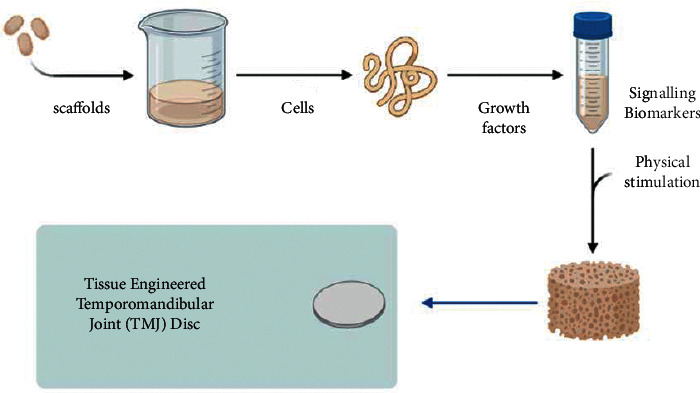
Steps and components of tissue engineering the TMJ disc.

Apart from this, other factors such as platelet derived growth factor, epidermal growth factor, interleukin-1, and tumor necrosis factor alpha also have role in proliferation, production of collagen, and glycosaminoglycans (GAG) [61]. In a recent study, Chen et al. reported inhibition of TNF-*α*/Nf-*κ*B promoted fibrocartilage stem cell's chondrogenic potential [62]. Kang et al. in their study reported that TGF-*β*1 and IGF-1 induced increase in type 1 collagen and GAG synthesis and cells proliferation [63]. In a study by Wang et al., it was observed that the concentrated growth factor leads to repair of goat TMJ. It assisted in cell proliferation and induced tissue repair [64].  Figure 1 shows the steps and components of tissue engineering the TMJ disc.

### 3.3. Hurdles in Tissue Engineering of the TMJ Disc

**Figure 2:**
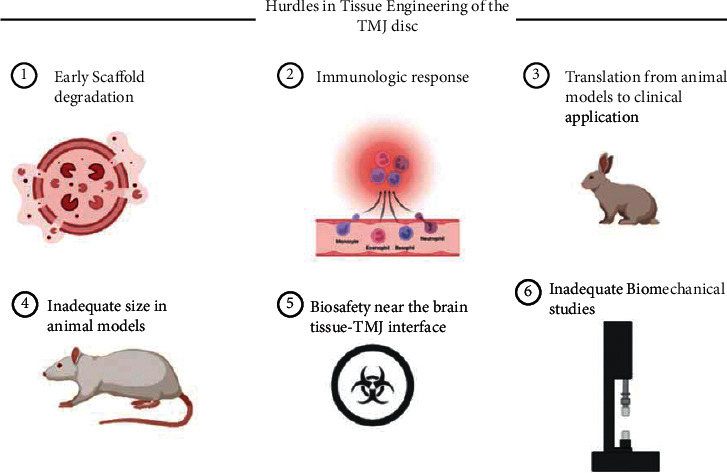
Hurdles in developing a practically useable TE TMJ disc.

Scaffolding material for TMJ disc TE remains a challenge because these materials exhibit differential contraction, insufficient mechanical strengths for load-bearing, degradation of the products, stress-shielding, and material-induced immunologic responses [31]. Novel techniques like nanoassembly and nanocoating are yet to be studied in detail, and self-assembling scaffold-free approaches have yet to be studied widely in TMJ disc regeneration [27, 65]. Furthermore, preclinical studies on smaller animal models fail to scale to the size and function of human TMJ, thereby limiting the application of smaller constructs in reparative surgeries of small disc perforation and thinning, but exclude their translation to an implantable disc after a discectomy or larger defects [66, 67]. In contrast to the knee joint, a complete understanding of TMJ biomechanics is still lacking to support preclinical studies. Treatment guidelines and studies specific to TMJ should bring forward additional knowledge of TMJ to levels of other diarthrodial joints like the knee [68]. Additionally, the proximity of the TMJ to the brain is another issue where an engineered disc will have to go through stringent scrutiny compared to TE implants for the knee joint which is not closely approximated to a high-stake tissue as the brain [69]. Caution must be exercised to ensure strict safety with regards to the brain when translating the engineered implantable disc from preclinical studies to clinical applications and to not repeat the conundrum associated with alloplastic Teflon-Proplast implants of the yesteryears [70, 71].  Figure 2 shows the hurdles in developing a practically useable TE TMJ disc.

### 3.4. Recent Advances and Future Directions

**Figure 3:**
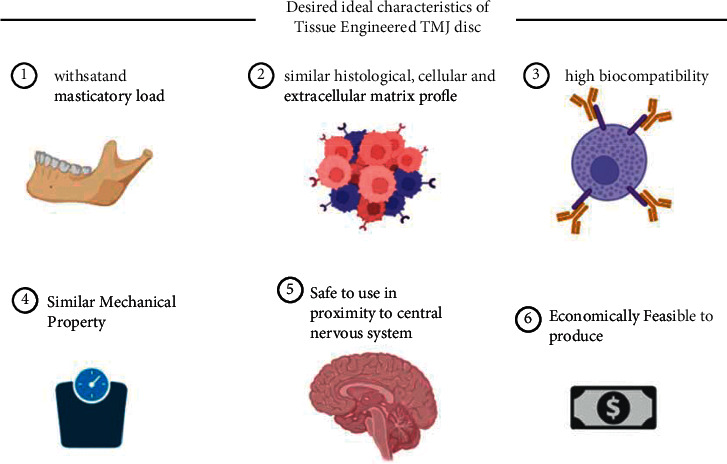
Ideal desired characteristics of a TE TMJ disc.

In a recent study on minipigs, TE discal implants were compared to empty controls. The tissue engineered discal implants restored “disc integrity by inducing 4.4 times more complete defect closure, formed 3.4-fold stiffer repair tissue, and promoted 3.2-fold stiffer intralaminar fusion. The osteoarthritis score (indicative of degenerative changes) of the untreated group was 3.0-fold of the implant-treated group.” These encouraging findings support that theoretically TE TMJ discs can one day supplant current grafts and alloplasts as the most convenient and effective treatments [72]. Bioprinting TMJ disc cells along with the ECM components representing the native TMJ disc with critical biosignaling molecules incorporated into the printed scaffolds may mimic the biomechanics and functions of the native disc. This combination of 3d bioprinting, appropriate signal markers, and exogenous mechanical stimulation could be the future in completing the advanced biomimicry required to end up with an engineered disc closer in function to the replaced disc. Self-assembling biochemical stimuli along with exogenous mechanical stimuli can be used to augment the mechanical properties of engineered discs to withstand the dynamic force in vivo. Scaling up of animal models closer to human TMJ is essential, and ensuring adequate thickness and size of the preclinical implants in animal models is paramount.  Figure 3 shows the ideal desired characteristics of a TE TMJ disc.

## 4. Conclusion

An ideal TMJ disc implant should have a few desirable properties; first, it should be able to withstand masticatory functional loads for a considerable long period or loading cycles; second, it should have a similar histological, cellular, and extracellular matrix profile, should have high biocompatibility and feasible to produce, and have familiar mechanical properties of the native disc. Current limitations of tissue engineering strategies for implantable TMJ disc analogue are not near completion; nevertheless, they represent a promising future in the disc replacement strategies for the clinicians. Safety issues related to immunologic response towards the brain tissue is critical in formulating indications for the application of TE TMJ disc in clinical practice.
